# The power of precision medicine: tailoring success in mitral annular calcification

**DOI:** 10.1093/ehjcr/ytae279

**Published:** 2024-06-06

**Authors:** Catarina Oliveira, João Silva Marques, Fausto J Pinto

**Affiliations:** Serviço de Cardiologia, Departamento de Coração e Vasos, ULS Santa Maria, Av Prof. Egas Moniz, 1649-028 Lisboa, Portugal; Serviço de Cardiologia, Departamento de Coração e Vasos, ULS Santa Maria, Av Prof. Egas Moniz, 1649-028 Lisboa, Portugal; Structural and Coronary Heart Disease Unit, Centro Cardiovascular da Universidade de Lisboa (CCUL@RISE), Faculdade de Medicina, Universidade de Lisboa, Av Prof. Egas Moniz, 1649-028 Lisboa, Portugal; Serviço de Cardiologia, Departamento de Coração e Vasos, ULS Santa Maria, Av Prof. Egas Moniz, 1649-028 Lisboa, Portugal; Structural and Coronary Heart Disease Unit, Centro Cardiovascular da Universidade de Lisboa (CCUL@RISE), Faculdade de Medicina, Universidade de Lisboa, Av Prof. Egas Moniz, 1649-028 Lisboa, Portugal

**Figure:**
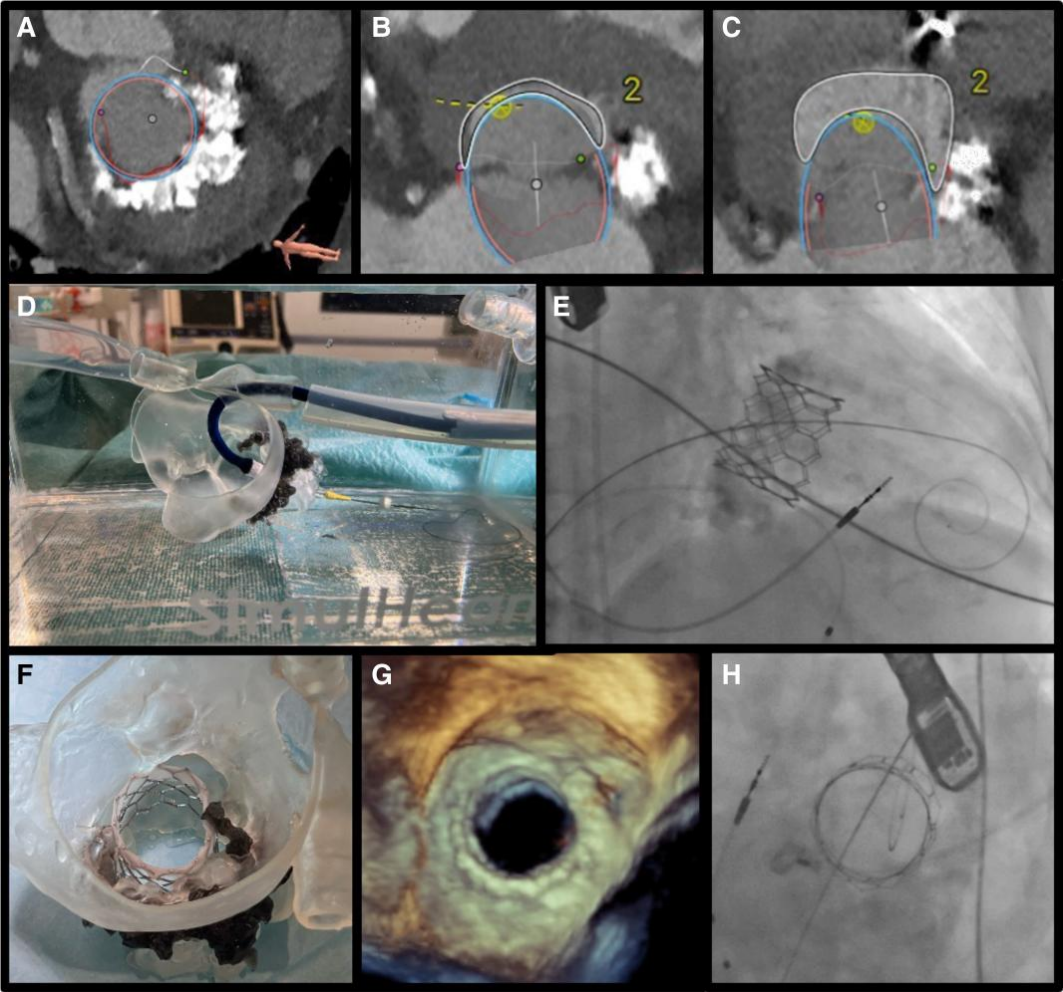


An 81-year-old woman with heart failure with normal ejection fraction was diagnosed with mitral annular calcification (MAC) causing moderate–severe mitral disease. Heart team decision was to evaluate candidacy for a valve-in-MAC (ViMAC) procedure due to high anatomic and surgical risk (STS score 12.9%, Euroscore II 5.36%). Pre-procedural CT showed feasibility for ViMAC based on mitral annular area and a MAC score of 8 (*Panel A*), but predicted a significant risk of left ventricular outflow tract (LVOT) obstruction with a neoLVOT area of 129 mm^2^ (*Panel B*). To address this, alcohol septal ablation (ASA) was performed. Follow-up CT showed a substantial increase in neoLVOT area to 411 mm^2^, strikingly reducing the obstruction risk (*Panel C*).

For procedural planning, a novel approach utilizing patient-specific simulation and multimaterial 3D printing was adopted to replicate cardiac anatomy. This allowed successful implantation of a 29 mm SAPIEN 3 valve, closely mirroring the steps needed in the actual procedure (*Panel D*). This precision medicine approach was further pursued by briefing the interventional team on every critical step of the procedure and bailout options. This resulted in a smooth procedure with implantation of the 29 mm SAPIEN 3 valve under fluoroscopic and transoesophageal echo guidance, as predicted by simulation (*Panels E*–*H*). The patient’s post-procedural recovery was excellent, with significant symptom improvement and no hospital readmissions.

This case exemplifies the importance of ASA in preventing LVOT obstruction during transcatheter mitral valve replacements and showcases a custom-tailored procedural planning method, highlighting the potential of precision medicine in complex cardiac interventions.

**Consent:** The patient provided written consent for the use of all data to academic and research purposes as well as for publication.

**Funding:** None declared.

## Data Availability

All data are available upon request.

